# Clock advance and magnitude limitation through fault interaction: the case of the 2016 central Italy earthquake sequence

**DOI:** 10.1038/s41598-019-41453-1

**Published:** 2019-03-21

**Authors:** Nicola Alessandro Pino, Vincenzo Convertito, Raul Madariaga

**Affiliations:** 1 Istituto Nazionale di Geofisica e Vulcanologia, Osservatorio Vesuviano, Via Diocleziano, 328, 80134 Naples, Italy; 2 Ecole Normale Supérieure, Laboratoire de Géologie, 24 rue Lhomond, 75231 Paris cedex 05, France

## Abstract

Faults communicate with each other. Strong earthquakes perturb stress over large volumes modifying the load on nearby faults and their resistance to slip. The causative fault induces permanent or transient perturbations that can change the time to the next seismic rupture with respect to that expected for a steadily accumulating stress. For a given fault, an increase of stress or a strength decrease would drive it closer to - or maybe even trigger - an earthquake. This is usually perceived as an undesired circumstance. However, with respect to the potential damage, a time advance might not necessarily be a bad thing. Here we show that the central Italy seismic sequence starting with the Amatrice earthquake on 24 August 2016 advanced the 30 October Norcia earthquake (M_W_ = 6.5), but limited its magnitude by inhibiting the rupture on large portions of the fault plane. The preceding events hastened the mainshock and determined its features by shaping a patch of concentrated stress. During the Norcia earthquake, the coseismic slip remained substantially confined to this patch. Our results demonstrate that monitoring the seismicity with very dense networks and timely analyses can make it feasible to map rupture prone areas.

## Introduction

Plate motions cause build up of stress on faults during decades or centuries, which is released during large earthquakes. Seismic events with magnitude M_W_ above 5.8–6.0 on average are associated with fault length larger than about 10 km (ref.^1^), with typical slips of the order of 20 cm (refs^2,3^), inducing significant strain in the neighbouring area. Static changes of stress field and fault strength result from such large strains, which also induce dynamic effects connected with viscous relaxation of the lower crust and diffusive processes associated with flow of crustal fluids. According to the amount and the sign of the previous level of stress, and to the changes caused by the earthquake, these permanent and temporary processes result in shadow zones where the rupture is inhibited and areas where the potential for earthquake nucleation is enhanced, thus advancing the failure^4–7^.

In the last 20 years, a large number of studies have been published analysing the variation of the stress field produced by one or more earthquakes in the nearby volume (e.g., refs^8–10^). When dealing with some specific receiver fault where a new failure was triggered, most investigations mainly focused on the location of the hypocenter with respect to the areas of increased load stress on the fault^11,12^ and only in a few cases the analysis considers the full slip distribution on the receiving fault (e.g., ref.^13^).

However, given that the cumulative stress field following an event can vary over relatively short wavelengths, strong stress and strength heterogeneity may develop on extended nearby faults and create conditions for earthquake complexity by controlling seismic rupture start, growth, and termination. This means that time shift for earthquakes (i.e., change in the time to the next rupture with respect to that expected for a steadily accumulating stress) could be associated with stress increase or decrease on different areas of its fault plane, reshaping the patches where stress is concentrated (asperities) and significantly modifying the energy available for seismic rupture and radiation in a future event. In this framework, mapping the seismicity in space and time and the stress changes caused by a seismic event on nearby existing faults may provide us with images of the preparation toward the next failure, allowing estimation of the areas prone to dislocate and their potential radiation, i.e. the event size.

**Figure 1:**
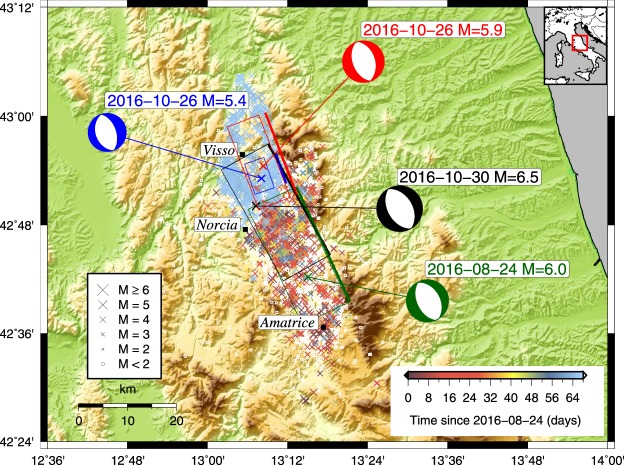
Seismicity map of the Amatrice seismic sequence. Epicentral location of the earthquakes occurring since 24 August 2016 to the time of the 30 October 2016, Norcia earthquake^29^. The symbol colour and size change according to time of occurrence and magnitude (except for the M < 2 events, all displayed in white colour, and for the mainshocks to preserve clarity), respectively, while the analysed events are indicated by green, blue, red, and black crosses. The fault mechanisms^14^ (Supplementary Table 1) of the largest events are also displayed as beachballs, using the same colour as the location. The black rectangles represent the surface projection of the fault planes (P1 in Supplementary Table 1), as inferred from both the focal mechanisms and surface displacements^29,30^. For each plane, the intersection with the free surface is depicted by a thick line of the same colour.

We investigate the preparatory process of the 30 October 2016 Norcia (Central Italy) earthquake (M_W_ = 6.5), by computing the stress changes caused on its causative fault by the strongest events in the preceding seismicity, starting with the initiation of the sequence on 24 August 2016. The sequence started with a M_W_ = 6.0 earthquake (Amatrice event), followed on 26 October by a pair of events (M_W_ = 5.4 and M_W_ = 5.9, Visso events) located between 22 and 25 km north of Amatrice and, 4 days later, by the Norcia earthquake, which nucleated approximately in the middle of the elongated area spanned by the sequence (see Fig. 1 and Supplementary Table 1).

## Results

**Figure 2:**
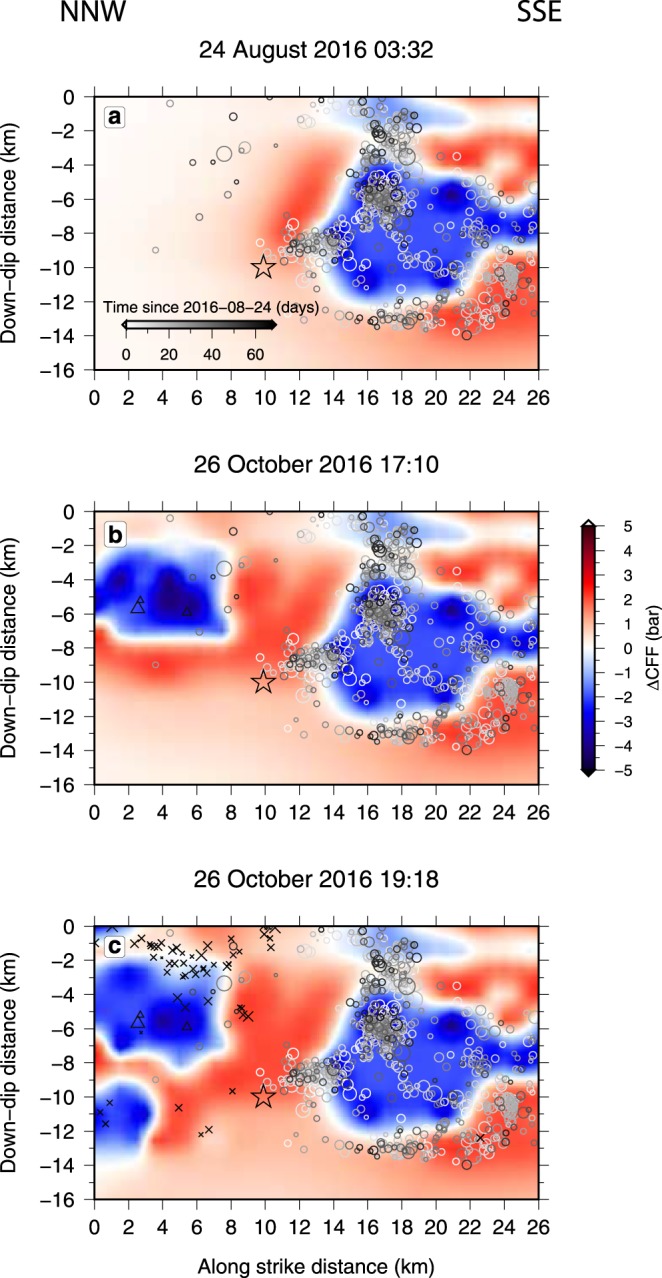
Coulomb failure function change (*ΔCFF*) on the fault plane of the 30 October 2016, Norcia earthquake, caused by the 3 strongest preceding events in the Amatrice seismic sequence, together with the aftershocks distribution^29^. (**a**) *ΔCFF* caused by the Amatrice 24 August 2016 event, along with the aftershocks (circles) occurring within 350 m (see Methods section) from the Norcia fault plane and up to 26 October 2016 17:10. Aftershocks are colour coded based on their origin time since 24 August 2016 (see time line in Fig. 2a). Positive and negative variations indicate respectively increased and decreased *ΔCFF* areas. (**b**) Same as (**a**), with the addition of the *ΔCFF* contribution of the 26 October 2016 17:10 and aftershocks (triangles) up to 26 October 19:18 UTC. The evident invariance of the *ΔCFF* in the southern half of the fault plane in the ~60 days time period indicates the negligible effect of viscous relaxation in the lower crust. (**c**) Same as (**b**), with the addition of the *ΔCFF* contribution of the 26 October 2016 19:18 UTC and aftershocks (crosses) up to 30 October 2016. In all the panels, the black empty star corresponds to the location of the Norcia event hypocenter^29^ (rupture nucleation).

We calculated the modification in the stress field on the fault plane of the Norcia earthquake^14^ (P1 in Supplementary Table 1), in terms of Coulomb failure function change (*ΔCFF*), relative to three subsequent time periods corresponding to the origin time of the largest events in the sequence (Fig. 2). All the analysed earthquakes are almost pure normal fault events^14^ (Supplementary Table 1). Differently from other authors^7,15^ we both use detailed slip distributions for all the 3 major events and include the effect of the viscous relaxation in the lower crust. The rupture associated with the Amatrice earthquake (M_W_ = 6.0) started 18 km south of the hypocenter of the Norcia earthquake and propagated northward. It induced on the fault plane that would subsequently rupture on the 30 October 2016 significant *CFF* changes that inhibited the rupture on the southern half of the plane – possibly limiting the available surface for the next breaking – and slightly increased the stress elsewhere (Fig. 2a). The aftershocks of this first earthquake were mainly peripheral to the reduced Coulomb stress area. Incidentally, we notice that assuming planar fault surfaces may slightly affect the extension of the *ΔCFF* areas and the relative position of the aftershocks. In the immediacy of the event, the dynamic strain associated with the radiated wavefield also contributed to trigger aftershocks^16^. The seismicity immediately following large earthquakes in the central Apennines is known to be affected by pore pressure waves generated by the mainshock^17–19^, by lowering the effective normal stress and favouring the slip. Nevertheless, these effects do not appear to overcome the *CFF* reduction produced on the southern half of the fault plane that ruptured to generate the Norcia earthquake on 30 October. Although in the two months following the Amatrice earthquake the aftershock area extended northward considerably – with numerous events occurring north of Norcia – the seismicity on this plane remained substantially confined to its southern portion, indicating the possible delineation of an asperity situated close to the central segment.

Following the Amatrice earthquake, two Visso earthquakes occurred on 26 October about 10 kilometres beyond Norcia (Fig. 1). Although these events are located in the area of increased coseismic stress and in the direction of higher dynamic strain due to northward rupture propagation, the ~60 days delay rule out instantaneous static or dynamic triggering produced by the 24 August event. On the other hand, the time difference is too short to allow for a viscous stress transfer through the lower crust to really make a difference (Fig. 2a,b). In fact, at the considered time scale and at distance range, and for the assumed rheological model, viscous effects can produce stress variation of the order of 0.1 bar (see also ref.^20^), significantly lower than the static *CFF* change (Fig. 2). Instead, the northward evolution of the whole sequence appears to be consistent with a diffusive process, associated with fluid flow induced in the upper crust^21,22^ by the 24 August Amatrice earthquake (Supplementary Fig. 1). A similar result has been obtained by other researchers^15,23^. However, if this is the case, fluids must have been going first through the Norcia fault, located south of the two 26 October events, but apparently the associated reduction of normal stress was not intense enough to trigger the rupture of this fault.

The 26 October Visso events also produced a strong stress decrease on the 30 October fault, but was limited to the northern area (Fig. 2b,c). Although significantly less numerous, again the aftershocks mainly concentrated at the border of the decreased stress area, with no events in the central segment of the fault.

At this time, the preceding seismicity created a very heterogeneous load pattern on the Norcia fault plane, shaping a well defined area of concentrated stress with no seismic events inside and bordered by clusters of aftershock hypocenters distributed along a roughly annular zone. These clusters are associated with relatively high *b*-value (low differential stress) toward the inner part of the asperity, indicating an “encircling maneuver” (ref.^24^) of the aftershocks, i.e., a gradual rupture of the asperity, first around its edge and then inward. Moreover, as expected^25,26^, the deeper cluster is characterised by significantly lower *b*-values, identifying the zone where the rupture nucleation is more likely to occur (Supplementary Fig. 2).

Thus, the previous earthquakes both increased the stress in the central portion of the fault and weakened the contour of this asperity – through stress corrosion enhanced by fluids^27^ – likely advancing the clock for the next failure. At the same time, the previous Amatrice and Visso earthquakes respectively on the southern and northern portions of the fault, limited the size of the area available for fracturing.

**Figure 3:**
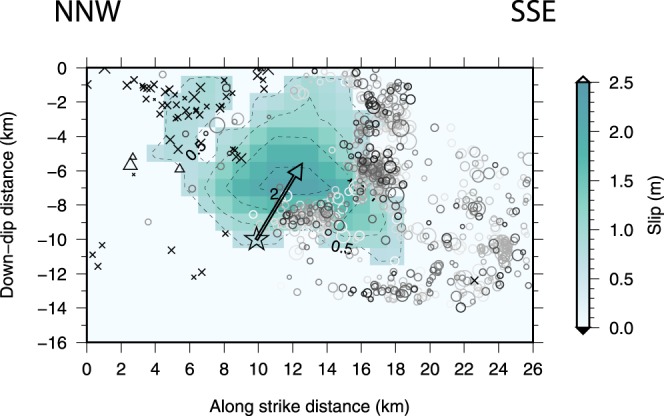
Dislocation associated with rupture of the Norcia 30 October 2016 earthquake^29,30^ (see Methods). The preceding seismicity^29^ since the 24 August 2016 Amatrice earthquake and occurring within 350 m (see Methods section) from the fault plane is also displayed. The colour code for time and symbols for aftershocks are the same as in Fig. 2. The foreshocks are distributed around the Norcia slip area – showing the “encircling maneuver” (ref.^24^) leading to the breakage of the asperity – and are clustered in three main patches, with varying *b*-value representing a complex pattern of the differential stress, increasing down-dip and away from the asperity (Supplementary Fig. 2). The empty star corresponds to the location of the Norcia event hypocenter^29^ (rupture nucleation), while the arrow indicates the dominant direction of rupture propagation.

Finally, four days later the rupture started in a positive Coulomb stress change area, propagating upward and destroying the asperity (Fig. 3). Notably, coseismic slip is strikingly complementary to the area broken by the preceding seismicity, with some slip in between two well defined clusters of aftershocks (Fig. 3: 8–10 km downdip; 14–18 km along strike). Beyond the nucleation zone, rupture did not have sufficient energy to penetrate the unloaded patches. The seismic moment corresponds to magnitude M_W_ = 6.5 (*M*_0_ = 7.07 × 10^18^ Nm). Based on constraints derived from surface geology and aftershocks’ location, the whole surface represented in Fig. 3 – corresponding to the Mt. Vettore-Mt. Bove structure – constitutes a single 31 km-long seismogenic source, with total potential rupture area of ~440 km^2^ (refs^7,28^). This area is about twice the area that ruptured on the 30 October. Thus, if the rupture involved the entire Mt. Vettore-Mt. Bove structure, the eventual total seismic moment would have been double at least, if the same average slip is cautiously assumed, corresponding to a magnitude M_W_ = 6.7.

Besides, by considering the fault models and slip distributions adopted in the present analysis^29,30^ – not including slip on multiple segments, as suggested by other authors (e.g., ref.^15^) – we notice that in spite of the definitely larger seismic moment *M*_0_ − 7.07 × 10^18^ Nm against 1.07 × 10^18^ (ref.^14^) – the final displaced area of the Norcia earthquake was comparable to that of the Amatrice earthquake, but the maximum slip was more than twice as large. These ratios do not correspond to what is predicted by empirical scaling relations for seismic moment *M*_0_, fault length *L* and width *W*, and maximum slip *Δu* (*Δu∝L*; *M*_0_ = *∝L*^3^ *or M*_0_ = *∝L*^2^*W)* (ref.^31^), expected to be satisfied by earthquakes occurring in the same area. These relations would require some proportionality between the rupture surface and the maximum slip.

In order to estimate the time (*ΔT*) it would have taken for the stress imposed by the major previous events in the sequence to have accumulated naturally, we divide the *ΔCFF* = 1.13 bar estimated at the Norcia earthquake hypocentre by the Mt. Vettore fault stress-loading rate of 0.0028 bar/yr – modelled by using historical earthquakes on active faults in the area^7^ – giving *ΔT*~400 yr. This means that the Norcia event would have occurred anyway in year 2016 + X, where X ≤ 400 yr representing the actual time advance, but the preceding earthquakes in the 2016 sequence made it happen. By assuming for the Mt. Vettore fault both the time of the last earthquake (500 A.D.) and the recurrence time (1627 yr) used for time dependent seismic hazard computation^32^, X could be about 110 yr.

## Conclusion

Based on our analysis, we propose that the seismicity preceding the 30 October Norcia earthquake created the conditions that made this event to occur in advance. In the ruptured area the stress increase was not very large (of the order of a few bars) – significantly lower than both the apparent stress drop estimated for apenninic normal fault earthquakes (~30 bar; refs^33,34^) – meaning that stresses had been building up on this fault for several centuries^7,35^. In particular, the anomalously high static stress drop of the 30 October Norcia event (300 bar; ref.^36^) calls for a high energy release per unit area, further supporting the conclusion of a definitely small ruptured area, with respect to what expected from source scaling laws for apenninic events.

The delineation and the erosion of the asperity – possibly helped by pore pressure increase caused by fluid flow in the upper crust – raised the stress gradient and accelerated seismic rupture. Conversely, the previous events limited the available surface for breaking and thus the energy released during fracturing, both by determining clear patches of lowered Coulomb stress acting as stress shadows – in accordance with other studies^15^ – and by delineating a well defined asperity through the aftershocks’ distribution. Therefore, without the preparatory process accelerated by the preceding seismic sequence, the Norcia earthquake would have occurred later, but probably with a larger seismic moment. Our conclusion presents an additional view of the sequence evolution with respect to other authors (refs^15,37^), suggesting that structural segmentation controls the final rupture extent of the main events in the sequence.

We also notice that the foreshock pattern – with all the previous events distributed around the patch that would break subsequently on the 30 October – is compatible with the cascade model of rupture nucleation, rather than the pre-slip model characterized by slow slip and small events inside the asperity^38^.

In this framework, we believe that in addition to the estimate of the long-term tectonic stress load and of reliable slip distribution of previous nearby earthquakes, together with their associated stress changes, precise and timely mapping of the seismicity could provide us with valuable information about the seismic potential of known seismogenic structures. This means that a significant effort should be put forward in the higher seismic hazard regions to map the active faults and their seismicity.

## Methods

### Coulomb stress change

We compute the Coulomb stress changes caused on the fault plane of the 30 October 2016 (Norcia) earthquake by the three largest events from 24 August 2016 through 26 October 2016. Then we compare the results with both the seismicity since the 24 August 2016 Amatrice earthquake and up to 30 October 2016, occurring within 350 m from the fault plane, and the slip distribution of the Norcia earthquake. The limit of 350 m was chosen taking in to account the distribution of aftershock location error, whose modal value is lower than 0.1 km for horizontal location and lower than 0.5 km for vertical location (given the dip of the fault plane, nearly all the aftershocks occurring on the fault plane are thus included). We obtained the slip distribution as the geometric mean of the distribution derived from waveform inversion^29^ and the one inferred from surface deformation data (ref.^30^, their figure S11b). We removed slip less than 20% of the maximum value, in order to remove unstable, less constrained model’s features and preserve the features common to both geodetic and seismological slip distributions.

Coherently with the fault geometry used to infer the slip models^29,30^, in our computation we assume planar models for both causative and receiving faults. Potential azimuthal variations along the actual fault surfaces might result in different Coulomb stress change with respect to what obtained for planar faults. However, we are interested at the gross picture and the surface data (geodetic measurements and field detected surface breakage) indicate that azimuthal variation can possibly occur at smaller scale than that of our analysis.

We calculated the co- and post-seismic deformation and the associated Coulomb stress change *ΔCFF* = *Δ*τ + *μ*(*Δ*σ + *Δp*), where *Δ*τ and *Δ*σ are respectively the shear and normal stress change, *μ* is the friction coefficient and *Δp* is the pore pressure change. We use a computer code based on the viscoelastic-gravitational dislocation theory^39^ and assume that *Δp* = 0, corresponding to drained conditions. The method allows the use of finite source fault models, with heterogeneous slip distribution. It uses the standard linear solid rheology defined by three parameters: the unrelaxed shear modulus *μ*_0_, the viscosity *η* and the parameter *α*, which is the ratio of the fully relaxed modulus to the unrelaxed modulus. As a difference with usual analyses, that consider the location of the nucleation of the following earthquakes relative to the induced stress variation, here we investigate the heterogeneity of the stress field on the whole fault plane and the time evolution of the earthquake preparatory process.

We adopt a 7-layered, viscous structural model (Supplementary Table 2), obtained by merging information from several published studies on the central Apennines crustal structure^40–44^, and the fault mechanisms retrieved from seismic waveform inversion^14^ (Supplementary Table 1). We assumed the causative fault plane on the basis of geodetic and seismological investigations and compute Green functions for a 90-day time window, each event contributing to the stress field from its origin time. We use 100 equally spaced horizontal points, on a distance range of 0–150 km, and 100 points in depth, ranging between 0 and 151 km. Stress field variation is computed on 7 different layers with depth ranging between 0 and 16 km.

For the 24 August 2016 earthquake (Amatrice, M_W_ = 6.0), we assume the focal mechanism derived from waveform inversion for the moment tensor solution (Supplementary Table 1). We selected one heterogeneous slip model derived from seismograms^29^ and one obtained from surface deformation^30^ data, in order to consider solutions from independent data. We average the two slip distributions by computing the geometric mean, to retain the most robust patterns and to attenuate the unstable patches. The results are then used as input for the stress field computation.

Concerning the two 26 October earthquakes (Visso), for the first one (M_W_ = 5.4) we assume uniform slip on a rectangular fault with dimensions L × W = 6.5 × 5.25 km^2^, derived from empirical relations^1^, while for the second (M_W_ = 5.9) we adopt the slip distribution derived from seismic waveforms^29^, again selecting only the slip equal or larger than 20% of the maximum dislocation, to remove minor, less constrained, areas of the model.

Albeit the stress computation results depend on the adopted slip distribution derived from inversion procedures, generally providing non-unique solution, we consider that averaging distinct slip models preserves only the most stable features of each solution. This conclusion is further supported by other studies analysing the three main events (e.g., ref.^37^) and based on independent data, which display the same major slip patches as the ones considered here.

### Directivity analysis

For the 30 October 2016 Norcia earthquake, we compute the dominant rupture propagation direction (Fig. 3) by projecting on the fault plane the horizontal projection of the dominant rupture direction that, in turn, is obtained from the azimuthal distribution of peak ground velocity. We use a Bayesian inversion scheme^45^ that allows to infer the parameters of the directivity function *C*_*d*_ (ref.^46^) for a generic linear, horizontal bilateral rupture

1 $${C}_{d}=\frac{1}{2}\sqrt{\frac{{(1+e)}^{2}}{{(1-\alpha cos\vartheta )}^{2}}+\frac{{(1-e)}^{2}}{{(1+\alpha cos\vartheta )}^{2}}}\,$$

where *ϑ* is the angle between the ray leaving the source and the direction of rupture propagation *ϕ* (ref.^47^), and *α* is the Mach number, that is, the ratio between the rupture velocity *v*_*r*_ and the *S*-wave velocity. The parameter *e* = (2 *L*′ *− L*)*/L* is the percent unilateral rupture, where *L* is the total rupture length and *L*′ is the length of the dominant rupture^48^: *e* = 1 corresponds to a unilateral rupture, whereas *e* = 0 corresponds to a bilateral rupture. For the 30 October earthquake we obtained *e* = 0.6 ± 0.1, corresponding to a nearly unilateral rupture.

### *b*-value estimation

For *b*-value and Mc cross sections, we select earthquakes within 350 m (see above) from the fault plane, totalling 834 earthquakes, and use the software “zmap”^49^. Mc is estimated through maximum curvature technique with 0.1 bins in magnitude, whereas the *b*-value is obtained by using the maximum likelihood method^50^: *b* = *log*_10_*e/(<M>* *-Mmin)*. We adopted a grid 0.1 × 0.1 km^2^ and for each node the *b*-value is computed by selecting a minimum of 30 events within a radius of 1.5 km. All the figures in this paper are generated by using the Generic Mapping Tools (http://gmt.soest.hawaii.edu/)^51^.

## Supplementary information

Can clock advance limit the magnitude of impending earthquakes?
