# Supplementary material for: Clock advance and magnitude limitation through fault interaction: the case of the 2016 central Italy earthquake sequence
Source: Sci Rep. 2019 Mar 21;9:5005. doi: 10.1038/s41598-019-41453-1 (PMC6428860; doi:10.1038/s41598-019-41453-1)
Supplement: Supplementary file 1 — Can clock advance limit the magnitude of impending earthquakes? [file 41598_2019_41453_MOESM1_ESM.pdf]

Supplementary information

Clock advance and magnitude limitation through fault interaction: the case of the 2016 central Italy earthquake sequence

Authors:

[1]*Nicola Alessandro Pino*, [1]Vincenzo Convertito, and [2]Raul Madariaga*

Affiliations:

1. Istituto Nazionale di Geofisica e Vulcanologia, Osservatorio Vesuviano

Via Diocleziano, 328

80134, Naples, Italy

Nicola Alessandro Pino & Vincenzo Convertito

2. Ecole Normale Supérieure, Laboratoire de Géologie

24 rue Lhomond

75231 Paris cedex 05, France

Raul Madariaga

* Corresponding author

Supplementary Table 1 Fault plane solutions and moment magnitude (M_W) for the strongest earthquakes in the Amatrice 2016 seismic sequence[1].

Origin time (UTC)	M_W	Fault mechanism (strike, dip, rake)
24 August 2016<br>03:36:32	6.0	*P1* 155°, 49°, -87°<br>*P2* 331°, 41°, -93°
26 October 2016<br>17:10:36	5.4	*P1* 161°, 38°, -90°<br>*P2* 341°, 52°, -90°
26 October 2016<br>19:18:05	5.9	*P1* 159°, 47°, -93°<br>*P2* 344°, 43°, -87°
30 October 2016<br>06:40:17	6.5	*P1* 151°, 47°, -89°<br>*P2* 330°, 43°, -91°

P1 = Principal fault plane

P2 = Auxiliary fault plane

Supplementary Table 2 Structural and rheological model used to compute Coulomb stress change. The model is obtained by merging information from several crustal[2,3,4,5,6] and viscoelastic models[7].

Depth (km)	V_P(km/s)	V_S(km/s)	ρ(kg/m^3)	η_1 (Pa*s)	η_2 (Pa*s)	γ
0.0 - 1.5	3.75	2.14	2275	-0.1E+00	-0.1E+00	1.000
1.5 - 4.5	4.94	2.82	2486	-0.1E+00	-0.1E+00	1.000
4.5 - 7.5	6.01	3.44	2706	-0.1E+00	-0.1E+00	1.000
7.5 - 16.5	5.55	3.15	2609	-0.1E+00	-0.1E+00	1.000
16.5 - 29.5	5.88	3.36	2677	0.01E+19	-0.1E+00	0.800
29.5 - 43.5	7.10	4.00	3000	1.00E+19	-1.00E+00	0.800
43.5 - ∞	7.90	4.40	3276	1.00E+19	-1.00E+00	0.800

V_P = P-wave velocity

V_S = S-wave velocity

ρ = density

η_1 = transient viscosity

η_2 = steady-state viscosity

γ= ratio between the effective (μ) and the unrelaxed shear modulus μ_0

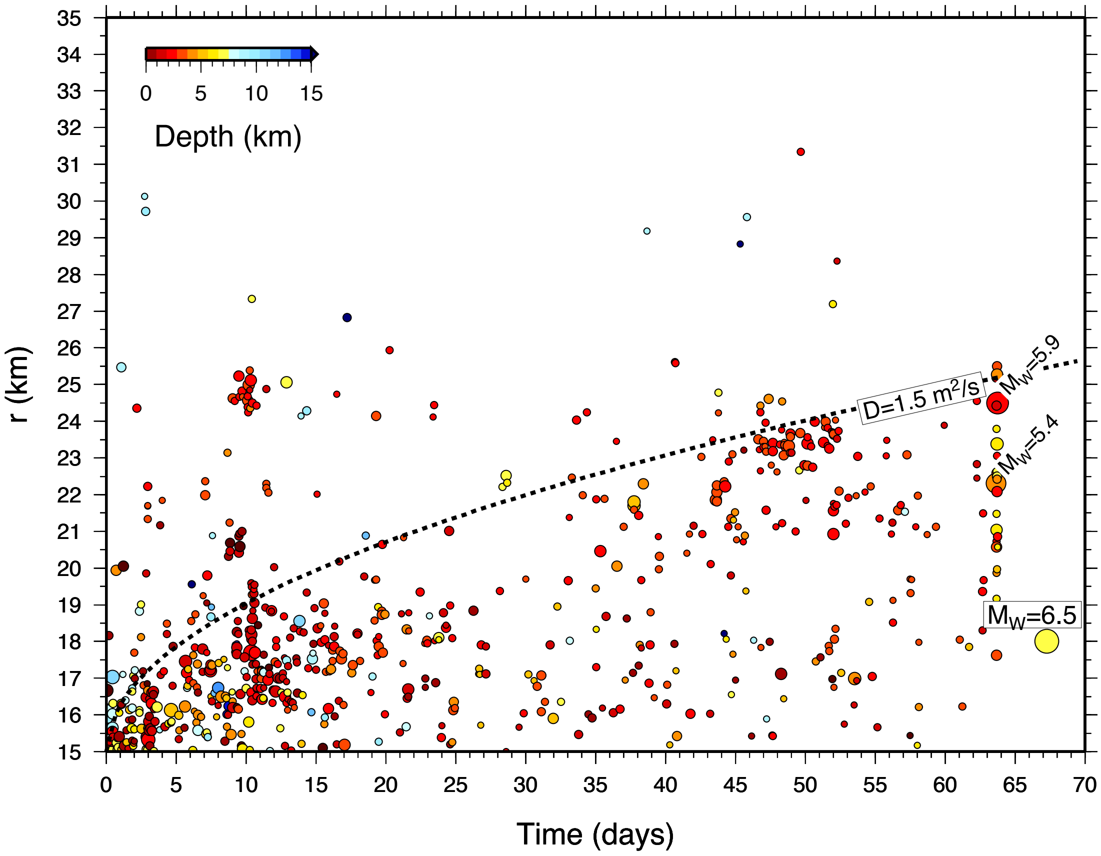

Supplementary Figure 1: A r-t (epicentral distance vs time) plot for the aftershocks occurred from the 24 August 2016 03:36:32, M_W=6.0, through the 26 October 2016, 19:18:05, M_W=5.9. The minimum M_W is 1.7, corresponding to the maximum completeness magnitude throughout the whole analysed period, except for the first 4 days from the beginning of the sequence[8]. The symbols are color coded according to the event hypocentral depth and scaled by magnitude. A minimum epicentral distance of 15 km is considered, to exclude the events on the fault plane of the 24 August earthquake and related to its rupture process. Dashed curve corresponds to the theoretical function $r=(4\pi Dt)^{1/2}$, computed for diffusivity values D=1.5 m^2/s (assumed homogeneous and isotropic), and represent the position of the fluid triggering front for a step-like pressure point source located at northern end of the 24 August 2016 03:36:32, M_W=6.0 fault. Events above the fluid triggering front correspond to earthquakes favoured by Coulomb static stress transfer or dynamic triggering[9]. The two 26 October Visso events and the 30 October Norcia event are also reported. The position of the M_W=6.5 Norcia suggests that its occurrence is not primarily promoted by the fluid diffusion process.

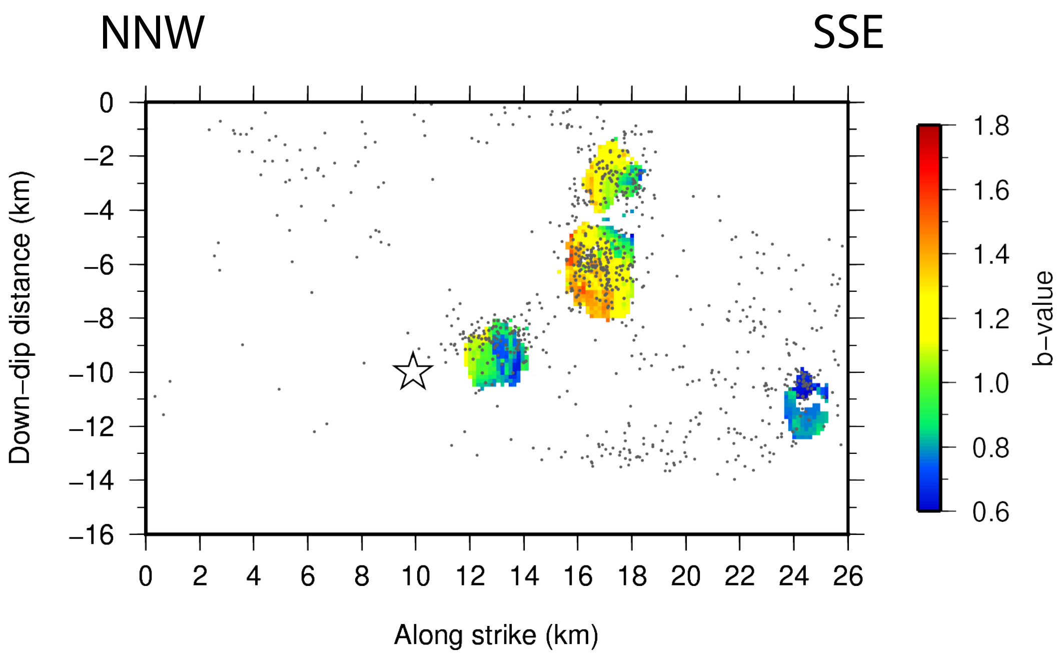

Supplementary Figure 2: *b*-value map computed on the fault plane of the 30 October 2016, Norcia earthquake, by using the events (black dots) located within 350 m (see Methods section) from the plane. The star indicates the hypocenter of the Norcia earthquake.

References

1. Istituto Nazionale di Geofisica e Vulcanologia (INGV) (last accessed on 15 October 2018). http://cnt.rm.ingv.it/tdmt

2. Patacca E., Scandone, P., Di Luzio, E., Cavinato, G. P. & Parotto, M., Structural architecture of the central Apennines: Interpretation of the CROP 11 seismic profile from the Adriatic coast to the orographic divide. *Tectonics* **27**, TC3006 (2008).

3. Di Stefano, R., Kissling, E., Chiarabba, C., Amato, A. & Giardini, D., Shallow subduction beneath Italy: Three-dimensional images of the Adriatic-European-Tyrrhenian lithosphere system based on high-quality P wave arrival times. *J. Geophys. Res.* **114**, B5305 (2009).

4. Scarascia, S., Lozej, A. & Cassinis, R., Crustal structures of the Ligurian, Tyrrhenian and Ionian seas and adjacent onshore areas interpreted from wide-angle seismic profiles. *Boll. Geof. Teor. Appl.* **36**, 5-20 (1994).

5. Di Luzio, E., Mele, G., Tiberti, M. M., Cavinato, G. P. & Parotto, M., Moho deepening and shallow upper crustal delamination beneath the central Apennines. *Earth Planet. Sci. Lett.* **280**, 1-12 (2009).

6. Piana Agostinetti, N. & Amato, A., Moho depth and Vp/Vs ratio in peninsular Italy from teleseismic receiver functions. *J. Geophys. Res.* **114**, B06303 (2009).

7. Cannelli, V., Melini, D. & Piersanti, A., Post-seismic stress relaxation with a linear transient rheology. *Ann. Geophys.* **53**, 89-99 (2010).

8. Chiaraluce, L. *et al.* The 2016 Central Italy seismic sequence: A first look at the mainshocks, aftershocks and source models. *Seismol. Res. Lett.* **88**, 757-771 (2017)

9. Convertito, V., De Matteis, R. & Pino, N. A., Evidence for static and dynamic triggering of seismicity following the 24 August 2016, M_W=6.0, Amatrice (central Italy) earthquake. *Pure Appl. Geoph.* **174**, 3663-3673 (2017).
